# Supplementary material for: Modeling glioblastoma heterogeneity as a dynamic network of cell states
Source: Mol Syst Biol. 2021 Sep 16;17(9):e10105. doi: 10.15252/msb.202010105 (PMC8444284; doi:10.15252/msb.202010105)
Supplement: Supplementary file 5 — Source Data for Figure 3 [file MSB-17-e10105-s001.zip › Figure3A_sourcedata/GSEA_3065/hallmarks_state5.GseaPreranked.1623416302988/HALLMARK_OXIDATIVE_PHOSPHORYLATION.html]

Details for gene set HALLMARK\_OXIDATIVE\_PHOSPHORYLATION[GSEA]

|  || Dataset | state5 |
| Phenotype | NoPhenotypeAvailable |
| Upregulated in class | na\_neg |
| GeneSet | HALLMARK\_OXIDATIVE\_PHOSPHORYLATION |
| Enrichment Score (ES) | -0.72083294 |
| Normalized Enrichment Score (NES) | -2.699268 |
| Nominal p-value | 0.0 |
| FDR q-value | 0.0 |
| FWER p-Value | 0.0 |
Table: GSEA Results Summary

  

Fig 1: Enrichment plot: HALLMARK\_OXIDATIVE\_PHOSPHORYLATION      
 Profile of the Running ES Score & Positions of GeneSet Members on the Rank Ordered List

  

| PROBE | GENE SYMBOL | GENE\_TITLE | RANK IN GENE LIST | RANK METRIC SCORE | RUNNING ES | CORE ENRICHMENT || 1 | POR |  |  | 215 | 0.430 | -0.0168 | No |
| 2 | ATP6AP1 |  |  | 288 | 0.388 | -0.0182 | No |
| 3 | ATP6V0B |  |  | 455 | 0.324 | -0.0313 | No |
| 4 | ATP1B1 |  |  | 548 | 0.298 | -0.0365 | No |
| 5 | RHOT2 |  |  | 660 | 0.273 | -0.0443 | No |
| 6 | NDUFC2 |  |  | 789 | 0.243 | -0.0546 | No |
| 7 | IDH1 |  |  | 793 | 0.242 | -0.0506 | No |
| 8 | NNT |  |  | 1212 | 0.183 | -0.0949 | No |
| 9 | ABCB7 |  |  | 1740 | 0.130 | -0.1525 | No |
| 10 | ATP6V1H |  |  | 2034 | 0.109 | -0.1839 | No |
| 11 | PMPCA |  |  | 2227 | 0.096 | -0.2040 | No |
| 12 | LRPPRC |  |  | 2329 | 0.090 | -0.2139 | No |
| 13 | SUPV3L1 |  |  | 2959 | 0.055 | -0.2844 | No |
| 14 | NDUFA9 |  |  | 3254 | 0.038 | -0.3172 | No |
| 15 | COX11 |  |  | 3309 | 0.036 | -0.3227 | No |
| 16 | DLST |  |  | 3524 | 0.026 | -0.3465 | No |
| 17 | MRPS11 |  |  | 3882 | 0.013 | -0.3869 | No |
| 18 | AFG3L2 |  |  | 3901 | 0.012 | -0.3887 | No |
| 19 | OGDH |  |  | 3946 | 0.009 | -0.3936 | No |
| 20 | IMMT |  |  | 3978 | 0.008 | -0.3970 | No |
| 21 | BDH2 |  |  | 4092 | 0.004 | -0.4097 | No |
| 22 | HADHA |  |  | 4438 | -0.012 | -0.4487 | No |
| 23 | SDHA |  |  | 4458 | -0.013 | -0.4507 | No |
| 24 | FXN |  |  | 4491 | -0.014 | -0.4541 | No |
| 25 | COX15 |  |  | 4728 | -0.023 | -0.4805 | No |
| 26 | ACO2 |  |  | 4811 | -0.026 | -0.4894 | No |
| 27 | MRPS22 |  |  | 4912 | -0.030 | -0.5002 | No |
| 28 | TOMM70 |  |  | 5004 | -0.033 | -0.5100 | No |
| 29 | OXA1L |  |  | 5079 | -0.036 | -0.5177 | No |
| 30 | GOT2 |  |  | 5108 | -0.037 | -0.5203 | No |
| 31 | PDHX |  |  | 5153 | -0.038 | -0.5246 | No |
| 32 | COX6A1 |  |  | 5281 | -0.043 | -0.5382 | No |
| 33 | RHOT1 |  |  | 5305 | -0.044 | -0.5401 | No |
| 34 | OPA1 |  |  | 5489 | -0.052 | -0.5600 | No |
| 35 | DLAT |  |  | 5529 | -0.054 | -0.5634 | No |
| 36 | MFN2 |  |  | 5580 | -0.057 | -0.5681 | No |
| 37 | MRPL35 |  |  | 5678 | -0.061 | -0.5781 | No |
| 38 | ALDH6A1 |  |  | 5682 | -0.061 | -0.5773 | No |
| 39 | NDUFS2 |  |  | 5820 | -0.066 | -0.5917 | No |
| 40 | MRPS30 |  |  | 5841 | -0.067 | -0.5928 | No |
| 41 | HTRA2 |  |  | 5885 | -0.069 | -0.5965 | No |
| 42 | IDH3G |  |  | 5901 | -0.070 | -0.5969 | No |
| 43 | NDUFS1 |  |  | 5905 | -0.070 | -0.5961 | No |
| 44 | CASP7 |  |  | 5942 | -0.072 | -0.5989 | No |
| 45 | ACAT1 |  |  | 6014 | -0.075 | -0.6056 | No |
| 46 | SUCLA2 |  |  | 6021 | -0.075 | -0.6050 | No |
| 47 | NDUFA7 |  |  | 6023 | -0.075 | -0.6038 | No |
| 48 | ACADVL |  |  | 6028 | -0.076 | -0.6029 | No |
| 49 | AIFM1 |  |  | 6081 | -0.078 | -0.6074 | No |
| 50 | MAOB |  |  | 6100 | -0.080 | -0.6081 | No |
| 51 | SDHD |  |  | 6111 | -0.080 | -0.6078 | No |
| 52 | ATP6V0E1 |  |  | 6138 | -0.082 | -0.6093 | No |
| 53 | HCCS |  |  | 6183 | -0.084 | -0.6128 | No |
| 54 | SDHC |  |  | 6193 | -0.085 | -0.6123 | No |
| 55 | CS |  |  | 6219 | -0.086 | -0.6137 | No |
| 56 | PHYH |  |  | 6255 | -0.088 | -0.6161 | No |
| 57 | ATP6V1C1 |  |  | 6336 | -0.092 | -0.6236 | No |
| 58 | IDH3A |  |  | 6585 | -0.107 | -0.6499 | No |
| 59 | OAT |  |  | 6648 | -0.111 | -0.6549 | No |
| 60 | IDH3B |  |  | 6850 | -0.125 | -0.6756 | No |
| 61 | ACAA1 |  |  | 6891 | -0.128 | -0.6779 | No |
| 62 | UQCRC2 |  |  | 7067 | -0.143 | -0.6952 | No |
| 63 | ALAS1 |  |  | 7091 | -0.144 | -0.6953 | No |
| 64 | HADHB |  |  | 7132 | -0.148 | -0.6972 | No |
| 65 | GRPEL1 |  |  | 7262 | -0.163 | -0.7090 | No |
| 66 | PDHA1 |  |  | 7277 | -0.165 | -0.7077 | No |
| 67 | DLD |  |  | 7363 | -0.176 | -0.7142 | No |
| 68 | MDH1 |  |  | 7422 | -0.184 | -0.7176 | Yes |
| 69 | NDUFB6 |  |  | 7433 | -0.185 | -0.7155 | Yes |
| 70 | TIMM9 |  |  | 7435 | -0.185 | -0.7123 | Yes |
| 71 | MTX2 |  |  | 7467 | -0.188 | -0.7125 | Yes |
| 72 | HSPA9 |  |  | 7469 | -0.188 | -0.7093 | Yes |
| 73 | TIMM17A |  |  | 7482 | -0.189 | -0.7073 | Yes |
| 74 | TIMM50 |  |  | 7527 | -0.194 | -0.7089 | Yes |
| 75 | NDUFS3 |  |  | 7530 | -0.195 | -0.7057 | Yes |
| 76 | GLUD1 |  |  | 7545 | -0.197 | -0.7038 | Yes |
| 77 | ACADM |  |  | 7551 | -0.198 | -0.7009 | Yes |
| 78 | NQO2 |  |  | 7580 | -0.201 | -0.7005 | Yes |
| 79 | PDHB |  |  | 7586 | -0.202 | -0.6975 | Yes |
| 80 | ETFA |  |  | 7588 | -0.202 | -0.6941 | Yes |
| 81 | ATP6V1D |  |  | 7635 | -0.208 | -0.6956 | Yes |
| 82 | NDUFS7 |  |  | 7658 | -0.211 | -0.6944 | Yes |
| 83 | MGST3 |  |  | 7746 | -0.225 | -0.7003 | Yes |
| 84 | NDUFV1 |  |  | 7768 | -0.228 | -0.6987 | Yes |
| 85 | PDP1 |  |  | 7777 | -0.229 | -0.6955 | Yes |
| 86 | FDX1 |  |  | 7788 | -0.232 | -0.6926 | Yes |
| 87 | SDHB |  |  | 7809 | -0.236 | -0.6907 | Yes |
| 88 | NDUFS4 |  |  | 7831 | -0.241 | -0.6888 | Yes |
| 89 | FH |  |  | 7851 | -0.243 | -0.6867 | Yes |
| 90 | ATP5PB |  |  | 7876 | -0.249 | -0.6850 | Yes |
| 91 | CYB5R3 |  |  | 7886 | -0.252 | -0.6816 | Yes |
| 92 | NDUFA4 |  |  | 7910 | -0.257 | -0.6796 | Yes |
| 93 | TIMM10 |  |  | 7925 | -0.261 | -0.6766 | Yes |
| 94 | SURF1 |  |  | 7943 | -0.264 | -0.6739 | Yes |
| 95 | MRPL11 |  |  | 7963 | -0.268 | -0.6713 | Yes |
| 96 | ATP5MC3 |  |  | 8001 | -0.276 | -0.6707 | Yes |
| 97 | PRDX3 |  |  | 8005 | -0.277 | -0.6661 | Yes |
| 98 | NDUFA1 |  |  | 8008 | -0.277 | -0.6614 | Yes |
| 99 | ACAA2 |  |  | 8017 | -0.279 | -0.6574 | Yes |
| 100 | GPI |  |  | 8020 | -0.280 | -0.6527 | Yes |
| 101 | DECR1 |  |  | 8034 | -0.282 | -0.6492 | Yes |
| 102 | MRPS12 |  |  | 8039 | -0.283 | -0.6447 | Yes |
| 103 | HSD17B10 |  |  | 8042 | -0.284 | -0.6399 | Yes |
| 104 | ISCA1 |  |  | 8068 | -0.290 | -0.6376 | Yes |
| 105 | NDUFB5 |  |  | 8071 | -0.291 | -0.6327 | Yes |
| 106 | NDUFA3 |  |  | 8073 | -0.291 | -0.6277 | Yes |
| 107 | CYB5A |  |  | 8086 | -0.294 | -0.6238 | Yes |
| 108 | ECI1 |  |  | 8099 | -0.297 | -0.6200 | Yes |
| 109 | VDAC1 |  |  | 8120 | -0.300 | -0.6169 | Yes |
| 110 | SLC25A4 |  |  | 8128 | -0.303 | -0.6124 | Yes |
| 111 | NDUFA5 |  |  | 8141 | -0.307 | -0.6083 | Yes |
| 112 | SLC25A11 |  |  | 8143 | -0.308 | -0.6030 | Yes |
| 113 | UQCRC1 |  |  | 8149 | -0.309 | -0.5981 | Yes |
| 114 | VDAC3 |  |  | 8151 | -0.309 | -0.5928 | Yes |
| 115 | CYC1 |  |  | 8152 | -0.309 | -0.5873 | Yes |
| 116 | NDUFB8 |  |  | 8159 | -0.312 | -0.5825 | Yes |
| 117 | MPC1 |  |  | 8166 | -0.316 | -0.5776 | Yes |
| 118 | ATP5MC1 |  |  | 8169 | -0.317 | -0.5722 | Yes |
| 119 | ISCU |  |  | 8183 | -0.321 | -0.5680 | Yes |
| 120 | MRPL15 |  |  | 8184 | -0.321 | -0.5623 | Yes |
| 121 | PHB2 |  |  | 8199 | -0.326 | -0.5582 | Yes |
| 122 | SUCLG1 |  |  | 8202 | -0.327 | -0.5526 | Yes |
| 123 | ECH1 |  |  | 8238 | -0.335 | -0.5507 | Yes |
| 124 | LDHA |  |  | 8263 | -0.343 | -0.5474 | Yes |
| 125 | COX4I1 |  |  | 8281 | -0.348 | -0.5431 | Yes |
| 126 | NDUFS8 |  |  | 8308 | -0.357 | -0.5398 | Yes |
| 127 | NDUFAB1 |  |  | 8333 | -0.363 | -0.5361 | Yes |
| 128 | LDHB |  |  | 8372 | -0.379 | -0.5337 | Yes |
| 129 | ECHS1 |  |  | 8411 | -0.391 | -0.5311 | Yes |
| 130 | ATP6V1E1 |  |  | 8419 | -0.394 | -0.5250 | Yes |
| 131 | BAX |  |  | 8426 | -0.398 | -0.5186 | Yes |
| 132 | COX7A2L |  |  | 8430 | -0.400 | -0.5119 | Yes |
| 133 | NDUFB3 |  |  | 8447 | -0.406 | -0.5065 | Yes |
| 134 | NDUFB1 |  |  | 8462 | -0.415 | -0.5008 | Yes |
| 135 | ATP5ME |  |  | 8481 | -0.424 | -0.4954 | Yes |
| 136 | IDH2 |  |  | 8498 | -0.434 | -0.4895 | Yes |
| 137 | TOMM22 |  |  | 8501 | -0.434 | -0.4821 | Yes |
| 138 | MRPS15 |  |  | 8516 | -0.442 | -0.4759 | Yes |
| 139 | COX17 |  |  | 8525 | -0.446 | -0.4689 | Yes |
| 140 | NDUFB7 |  |  | 8556 | -0.463 | -0.4641 | Yes |
| 141 | ATP5F1A |  |  | 8559 | -0.464 | -0.4562 | Yes |
| 142 | VDAC2 |  |  | 8564 | -0.466 | -0.4484 | Yes |
| 143 | UQCRB |  |  | 8566 | -0.467 | -0.4403 | Yes |
| 144 | TIMM8B |  |  | 8568 | -0.468 | -0.4321 | Yes |
| 145 | NDUFA2 |  |  | 8575 | -0.472 | -0.4245 | Yes |
| 146 | NDUFA8 |  |  | 8593 | -0.483 | -0.4179 | Yes |
| 147 | POLR2F |  |  | 8607 | -0.496 | -0.4106 | Yes |
| 148 | ETFB |  |  | 8610 | -0.499 | -0.4020 | Yes |
| 149 | COX7B |  |  | 8614 | -0.502 | -0.3935 | Yes |
| 150 | NDUFC1 |  |  | 8616 | -0.503 | -0.3847 | Yes |
| 151 | ATP5F1C |  |  | 8634 | -0.514 | -0.3776 | Yes |
| 152 | SLC25A3 |  |  | 8637 | -0.517 | -0.3687 | Yes |
| 153 | CYCS |  |  | 8640 | -0.519 | -0.3597 | Yes |
| 154 | COX5B |  |  | 8646 | -0.523 | -0.3511 | Yes |
| 155 | MRPL34 |  |  | 8651 | -0.527 | -0.3422 | Yes |
| 156 | ATP5MF |  |  | 8662 | -0.535 | -0.3339 | Yes |
| 157 | SLC25A5 |  |  | 8681 | -0.546 | -0.3263 | Yes |
| 158 | NDUFA6 |  |  | 8687 | -0.550 | -0.3171 | Yes |
| 159 | ATP5PD |  |  | 8711 | -0.574 | -0.3096 | Yes |
| 160 | NDUFV2 |  |  | 8717 | -0.581 | -0.2999 | Yes |
| 161 | UQCRFS1 |  |  | 8720 | -0.582 | -0.2899 | Yes |
| 162 | ATP5F1B |  |  | 8722 | -0.584 | -0.2797 | Yes |
| 163 | ATP5PF |  |  | 8732 | -0.593 | -0.2702 | Yes |
| 164 | MDH2 |  |  | 8733 | -0.593 | -0.2598 | Yes |
| 165 | TIMM13 |  |  | 8737 | -0.596 | -0.2496 | Yes |
| 166 | ATP5PO |  |  | 8747 | -0.609 | -0.2399 | Yes |
| 167 | ATP5F1D |  |  | 8760 | -0.620 | -0.2303 | Yes |
| 168 | ATP6V1G1 |  |  | 8766 | -0.625 | -0.2198 | Yes |
| 169 | COX6C |  |  | 8777 | -0.633 | -0.2097 | Yes |
| 170 | COX7A2 |  |  | 8779 | -0.635 | -0.1987 | Yes |
| 171 | COX5A |  |  | 8785 | -0.639 | -0.1879 | Yes |
| 172 | NDUFB2 |  |  | 8796 | -0.647 | -0.1776 | Yes |
| 173 | UQCR11 |  |  | 8804 | -0.655 | -0.1669 | Yes |
| 174 | NDUFB4 |  |  | 8810 | -0.659 | -0.1558 | Yes |
| 175 | SLC25A6 |  |  | 8817 | -0.667 | -0.1447 | Yes |
| 176 | ATP5MC2 |  |  | 8825 | -0.678 | -0.1335 | Yes |
| 177 | ATP5MG |  |  | 8826 | -0.681 | -0.1215 | Yes |
| 178 | UQCR10 |  |  | 8843 | -0.703 | -0.1109 | Yes |
| 179 | COX8A |  |  | 8862 | -0.724 | -0.1002 | Yes |
| 180 | COX6B1 |  |  | 8873 | -0.739 | -0.0883 | Yes |
| 181 | COX7C |  |  | 8882 | -0.748 | -0.0760 | Yes |
| 182 | ATP5F1E |  |  | 8884 | -0.751 | -0.0628 | Yes |
| 183 | UQCRQ |  |  | 8887 | -0.754 | -0.0497 | Yes |
| 184 | NDUFS6 |  |  | 8888 | -0.755 | -0.0364 | Yes |
| 185 | ATP6V1F |  |  | 8924 | -0.828 | -0.0257 | Yes |
| 186 | UQCRH |  |  | 8951 | -0.885 | -0.0131 | Yes |
| 187 | GPX4 |  |  | 8966 | -0.946 | 0.0020 | Yes |
Table: GSEA details [plain text format]

  

Fig 2: HALLMARK\_OXIDATIVE\_PHOSPHORYLATION: Random ES distribution      
 Gene set null distribution of ES for **HALLMARK\_OXIDATIVE\_PHOSPHORYLATION**
